# Relationship Between Academic Engagement and Social Media Addiction in Ecuadorian University Students

**DOI:** 10.3390/bs16030416

**Published:** 2026-03-12

**Authors:** Yosbanys Roque Herrera, Santiago Alonso García, Anabela del Rosario Criollo Criollo, Juan Antonio López Núñez

**Affiliations:** 1 Facultad de Ciencias, Escuela Superior Politécnica de Chimborazo, Riobamba 060155, Ecuador; 2 Departamento de Organización Escolar y Currículo, Universidad de Granada, 18071 Granada, Spain; salonsog@ugr.es (S.A.G.); juanlope@ugr.es (J.A.L.N.); 3 Hospital Provincial General Docente Riobamba, Universidad Nacional de Chimborazo, Riobamba 060111, Ecuador; ccanabela@hotmail.com

**Keywords:** academic engagement, health science students, higher education, social network addiction

## Abstract

The overuse of social media is a multidimensional phenomenon with the capacity to influence the academic environment. Thus, this study aimed to establish the relationship between social media addiction and academic engagement in university students. The research employed a quantitative approach, a non-experimental design, a correlational scope, and a cross-sectional analysis. The population comprised 1200 students (65.3% female) with an average age of 21.4 years from the Faculty of Health Sciences at the National University of Chimborazo, in Riobamba, Ecuador, during the first academic term of 2023. Data were collected using the Utrecht Academic Engagement Scale and the Social Media Addiction Questionnaire. A total of 95.8% of participants had sufficient academic engagement, and 93.7% had a medium level of social media addiction. There was a statistically significant (*p* < 0.01) negative correlation between the variables of academic engagement and social media addiction (including their respective dimensions), with a mild-to-moderate intensity, as indicated by Pearson r values ranging from −0.101 to −0.297. Significant associations were found between the social media used by participants and their connection frequency, and significant associations were also found between their primary use of social media and the main reason perceived by participants for controlling their use.

## 1. Introduction

The Internet has become the most widely used means of communication for human interaction. It is estimated that more than 95% of adolescents and young people between the ages of 14 and 24 prefer to use messaging apps to socialize, with the most prominent being those classified as social networks (Sigerson & Cheng, 2018). This phenomenon has sparked social concern about the overuse of these virtual resources due to their potential to cause health problems (Valencia Ortiz et al., 2021; Brailovskaia & Margraf, 2024). Since the advent of social media, there has been a sustained increase in the number of users, with more than 55% of the world’s population utilizing these platforms (Arteaga Araujo et al., 2022). The statistical report for April and June 2022 shows that the Facebook platform included 135 million user accounts worldwide aged between 13 and 19 (Soria & Villegas Villacrés, 2024).

Thus, the lack of limitations on using social media has led to the emergence of addictive behaviors, generating concern in the scientific community. The unintended consequences range from psychological disorders to social and economic problems (Yana Salluca et al., 2022). Although there are several definitions of the phenomenon of social media addiction, most authors agree on its excessive, uncontrolled, and obsessive use with negative consequences on the health and socioeconomic activities of those affected (Sujarwoto et al., 2023).

According to Rojas Jara et al. (2018), the study of the potential implications of social media addiction, its risk factors, manifestations, and possible consequences in the various dimensions of human beings is recognized as important. Likewise, Roncero Rodríguez et al. (2023) suggest that its inclusion as a pathology within the DSM-5 (Diagnostic and Statistical Manual of Mental Disorders, 5th ed.) and the ICD-11 (International Classification of Diseases, 11th ed.) will depend on the scientific evidence that can be gathered and systematized from reliable sources.

Among the potential health conditions caused by social media addiction are social isolation, distortion of perception between the real and digital world, sleep deprivation, a loss of interest in other activities, depression, family problems, low self-esteem, physiological problems, anxiety, withdrawal syndrome, school failure, eating disorders, and an increase in violent behavior, bullying, and cyberbullying, among others (Caner et al., 2022; Soria & Villegas Villacrés, 2024; Sümen & Evgin, 2021; Shafiq et al., 2023; Huang et al., 2023).

Social media addiction also affects social relationships in face-to-face activities, giving rise to a new phenomenon called phubbing, which occurs when someone ignores another person in real life by focusing all their attention on a mobile digital device (Ergün et al., 2023; Stănculescu & Griffiths, 2022). This phenomenon has been linked to difficulties in cognitive flexibility (Tanhan et al., 2024).

Valencia Ortiz et al. (2021) mention that this type of addiction can cause academic procrastination and negatively impact subjective well-being, academic performance, and school retention (Zhao, 2021), as well as the relationship between peers and teachers. The authors point out that high-intensity and high-frequency Internet browsing produces changes in neuronal connectivity as well as brain function and structure. Several manifestations allow us to identify the presence of addiction to social networks (Morales, 2020):

- Tolerance, which is the need to spend more time online.
- Withdrawal, which is the discomfort that occurs when the connection is interrupted.
- Using these networks for longer than intended.
- Being unable to stop using the Internet, even when one wants to.
- Excessive time spent on Internet-related activities.
- Not performing daily activities because of time spent connected to the networks.
- Using the Internet despite being aware of its harmful potential.

In contrast, commitment is a term derived from the Latin word “compromissium”, which implies fidelity in the face of responsibility for fulfilling an obligation, duty, promise, or task. Hence, academic engagement constitutes a psychological state marked by a student’s sense of belonging, demonstrating effort, involvement, participation, and eminent appreciation of the curricular and extracurricular educational process. This requires the individual to have a high intrinsic motivation for the corresponding activities and tasks, focusing on them in a concentrated manner and without sparing any effort (Ramos Vera et al., 2023; Álvarez Pérez et al., 2021; Torres Escobar & Botero, 2021).

At a psychological level, academic engagement manifests itself through three dimensions: behavioral, referring to the way in which the student adheres to institutionally established rules and norms and other self-imposed ones; emotional, which is reflected through the expression of the student’s feeling of belonging to their university and passion for the activities they carry out in it; and cognitive, related to the self-regulation that the learning process exerts when developing academic tasks (Torres Escobar & Botero, 2021). In university contexts, an appropriate level of academic engagement is associated with a student perception of high-quality professional training and institutional prestige, manifesting as positive academic satisfaction (Flunger et al., 2022; Arredondo Salas et al., 2022).

In the academic environment, the level of engagement has been positively related to different variables: motivation, satisfaction, self-efficacy, teaching quality, school adaptation, cognitive autonomy, family and teacher support, connection between subject and task, social integration with peers, emotional intelligence, a proactive attitude, positive emotions, and solid academic goals, among others (Flunger et al., 2022; Froment & de-Besa, 2022; Wang et al., 2021; Chen et al., 2021; Martin et al., 2021). Other research on adolescents in Europe reports that students with greater commitment have lower levels of academic stress compared to students who are not committed to their school activities (Oporto Alonso et al., 2022).

In a qualitative study on academic engagement, in which 56 students from the Bachelor’s Degree in Early Childhood Education at the National University of Río Cuarto, Argentina, participated, Rigo and Rovere (2021) found that autonomy and responsibility were activated during a planned task, increasing the assessment of self-management of learning at that time and the use of information and communications technologies as a tool to access knowledge.

In line with this, and with the aim of reducing dropout rates and/or academic failure, several researchers investigated actions aimed at increasing academic engagement. Hervás Torres et al. (2022) implemented a learning mentoring service program for university students who required it, observing an improvement in this variable at the end of the planned activities. Likewise, Galarraga et al. (2025) conducted a study based on community service learning, observing that these types of activities not only influenced the development of social engagement in students but also reinforced their academic engagement.

Loján Carrión et al. (2025) found that university students in educational sciences programs spend an average of 3.1 h per day on non-academic activities, such as social media and video games, which is negatively correlated with academic performance, even at moderate intensity. These authors established a relationship between academic procrastination and digital exposure time, reflecting difficulties with time management. They also found that students who use artificial intelligence as a support system reported decreased knowledge and class participation. These findings led to the conclusion that promoting the appropriate use of technology is essential to cultivate responsible habits that enhance learning and academic engagement.

The phenomenon of the sustained transition to online education, marked by the development of efficient digital learning platforms, the growth of students’ mastery of digital skills, and the implicit risk of misuse of various Internet resources, is the subject of recurring and thorough research due to its potential negative effect on academic engagement (Katz et al., 2021).

Thus, the following research question was posed: What is the relationship between social media addiction and academic engagement among students of the Faculty of Health Sciences of the National University of Chimborazo during the first academic period of 2023?

Consequently, this study aimed to establish the relationship between social media addiction and academic engagement among students at the Faculty of Health Sciences of the National University of Chimborazo, Riobamba, Ecuador, during the first academic term of 2023.

## 2. Materials and Methods

The research presented employed a quantitative approach, a non-experimental or observational design, and a correlational and cross-sectional scope. It took place during the first academic period of 2023.

### 2.1. Participants

The entire population of participants was included in the study, comprising 1200 students (65.3% female) between the ages of 18 and 27 (with an average age of 21.4 years) enrolled in the first through sixth semesters of training programs at the Faculty of Health Sciences, National University of Chimborazo, Ecuador.

The population characteristics were as follows:

- According to career: medicine, 243; clinical psychology, 181; nursing, 247; physical therapy, 165; clinical laboratory, 200; and dentistry, 164.
- According to semester: sixth, 235; fifth, 165; fourth, 117; third, 180; second, 255; and first, 248.

### 2.2. Tools

The instruments used for data collection were the Ultrecht Academic Engagement Scale (UWES-S-17) (Cruzat Aliaga, 2020) and the Social Media Addiction Questionnaire (ARS) (Escurra Mayaute & Salas Blas, 2014).

#### 2.2.1. UWES-S-17

The UWES-S-17 consists of 17 items (Cruzat Aliaga, 2020), which are assessed using a Likert scale with seven possible values: (0)—not at all, (1)—almost not at all, (2)—rarely, (3)—sometimes, (4)—quite a bit, (5)—frequently, and (6)—always; these items make it possible to measure the variable academic engagement and its three corresponding dimensions:

- Vigor, as reflected in items 1, 4, 8, 12, 15, and 17, corresponds to the will, persistence, mental resistance, and energy dedicated to the effort required by academic work, which involves facing the various complexities and challenges of the context and the tasks themselves. The scale value for this was established using the means of the scores of the corresponding items: very low (<2.18), low (2.18–3.20), average (3.21–4.80), high (4.81–5.65), and very high (>5.65).
- Dedication, as reflected in items 2, 5, 7, 10, and 13, refers to the level of intensity with which the student is involved in academic training, as well as the sense of enthusiasm, pride, meaning, and inspiration in overcoming the difficulties it imposes during the process. The scale value for this was established using the means of the scores of the corresponding items: very low (<1.61), low (1.61–3.00), average (3.01–4.90), high (4.91–5.79), and very high (>5.79).
- Absorption, using item values 3, 6, 9, 11, 14, and 16, is measured by the intensity of the student’s concentration during their academic self-preparation, in addition to considering the degree to which they focus on completing the task. The scale value for this was established using the means of the scores of the corresponding items: very low (<1.61), low (1.61–2.75), average (2.76–4.40), high (4.41–5.35), and very high (>5.35).

The scale value of global academic engagement was also determined through the mean scores of all items, as follows: very low (<1.94), low (1.94–3.06), average (3.07–4.66), high (4.67–5.53), and very high (>5.53). Furthermore, all students who scored in the low and very low categories were considered to have insufficient academic engagement.

The UWES-S-17 validation report indicated an adequate model fit, with the following figures: a CFI of 0.990 and an RMSEA of 0.048, along with an explained variance of 66.82%, according to the study conducted by Tristán Monrroy et al. (2021), which involved 223 students from the Autonomous University of San Luis Potosí. Furthermore, the authors of the present research established the existence of Cronbach’s alpha values of 0.762 to 0.868 and a general one of 0.917; the level of sampling adequacy and reliability of the results was very significant (*p* < 0.001).

#### 2.2.2. ARS

The ARS consists of two sections (Escurra Mayaute & Salas Blas, 2014). The general section explores variables related to the social networks used, how these social networks are used, the frequency of connection to social networks, and the causes of self-control regarding social network use. The other section measures addiction to social networks and the three dimensions and is composed of 24 items with a Likert scale of five values: (0)—never, (1)—rarely, (2)—sometimes, (3)—almost always, and (4)—always.

Dimensions of social media addiction:

- Items 2, 3, 5, 6, 7, 13, 15, 19, 22, and 23 measure obsession with the use of social networks relative to the dimension of an individual’s uncontrollable impulse to be connected to this Internet resource, which can develop into states of anxiety. The scale value for this was established using the summations of the scores of the corresponding items: low (<11), medium (11–30), and high (>30).
- Items 4, 11, 12, 14, 20, and 24 indicate a lack of personal control, specifically an insufficient capacity for self-regulation of compulsive behavior resulting from an uncontrollable need to connect with social networks. The scale value for this was established using the summations of the scores of the corresponding items: low (<7), medium (7–18), and high (>18).
- Items 1, 8, 9, 10, 16, 17, 18, and 21 indicate excessive use of social networks, characterized by the high intensity of this Internet service, which exceeds the person’s actual needs. The scale value for this was established using the summations of the scores of the corresponding items: low (<9), medium (9–24), and high (>24).

Likewise, the overall scale value of social media addiction was determined by summing all items, resulting in the following: low (<26), medium (26–72), and high (>72).

Condori Sinty et al. (2023) conducted a validation process for the ARS, yielding the following results: Aiken’s V of 0.996; a global Cronbach’s alpha of 0.88; Cronbach’s alpha values by dimension ranging from 0.843 to 0.941; and a Kaiser–Meyer–Olkin index of 0.95 overall. Considering the demonstrated validity of this instrument, Gamboa Melgar et al. (2022) used it as a reference to carry out a convergent statistical validation process. Furthermore, the authors of the present research established the existence of a Cronbach’s alpha value of 0.954 and a KMO of 0.966; the level of sampling adequacy and reliability of the results was very significant (*p* < 0.001).

### 2.3. Procedure

The study was conducted in accordance with the principles of scientific research ethics, including beneficence, autonomy, and non-maleficence. Participants expressed their willingness to participate by providing their informed consent after having read the document in question that was available prior to the application of the instrument. The corresponding institutional directors issued the necessary authorization for administering the instrument.

The instrument was administered online for individual completion; however, the research team visited each group by semester, year of enrollment, and career to explain the nature of the research, the instrument’s characteristics, and informed consent procedures. The research did not require sample selection, as all students in the aforementioned population participated.

### 2.4. Statistical Analysis

The use of SPSS software (version 23.0; IBM software, Armonk, NY, USA) facilitated the organization and subsequent processing of the obtained data. The results were strictly used for scientific and academic purposes. The variables studied were characterized using descriptive statistics (analysis of relative and absolute frequencies using crossed tables). In addition, inferential analyses were conducted to determine associations. The Pearson chi-square test was used for nominal qualitative variables attached to the ARS (social networks used by participants, connection frequency, main use of social networks, and main reason for controlling social media use) (Chango Pilamunga et al., 2024). Meanwhile, the Pearson correlation test (Ortiz Pinilla & Ortiz Rico, 2021; Fiallos, 2021) was applied to ordinal qualitative variables to measure the correlation between the levels of social media addiction and academic engagement (including their respective dimensions).

## 3. Results

Data processing allowed us to establish the predominance of students with sufficient academic engagement (95.8%). We observed that, among these students, the medium level of addiction to social networks predominated globally (93.7%) and varied by dimension (between 51.4% and 54.6%). However, excessive use of social networks was observed at a high level (60.2%). Furthermore, academic engagement was significantly associated with addiction to social networks and their respective dimensions, as determined by *p*-values < 0.05 using the χ^2^ Pearson test (Table 1).

When characterizing the level of academic engagement, it was found that only 4.2% of participants fell below the average level, which was the predominant level (40.5%) (Table 2).

Crossing the values of the levels of academic engagement with those of addiction to social networks revealed the following results (Table 2):

- High levels of social media obsession were most common among students with average academic engagement (23.3%). In comparison, the majority of students with high academic engagement had a medium level of social media obsession (20.2%).
- Likewise, the lack of control over social media use was most frequent among students with an average level of academic engagement (21.3%). In comparison, students with a high level of academic engagement predominated among those with a medium level of social media obsession (21.9%).
- However, among respondents with average or high levels of academic engagement, excessive social media use was prevalent at 29.5% and 21.2%, respectively.
- Meanwhile, among participants with average or high levels of academic engagement, the average level of the overall measurement of social media addiction predominated (38.8% and 33.1%, respectively).

In the vigor dimension, corresponding to the academic engagement variable, a higher frequency of the average level was observed (50.4%); among students with this characteristic, the following patterns predominated (Table 3):

- Medium and high levels of obsession with social media (24.0% and 26.4%, respectively), as well as a lack of control over social media use (25.2% and 24.6%, respectively).
- A high level of excessive use of social networks (35.7%) and a medium level of overall addiction to social networks (48.6%).

The data inherent to the dedication dimension of the academic engagement variable revealed a predominance of very high levels (37.1%) among the students involved in the research; meanwhile, 33.0% had average levels. Cross-referencing these data with those of the social media addiction variable and its dimensions revealed some specific characteristics (Table 4):

- A total of 25.0% of participants exhibited a very high level of dedication, along with a medium level of obsession with social media and a lack of control over their social media use.
- There was a predominance of individuals with an average level of dedication and a high level of excessive social network use (25.8%).
- A total of 34.8% of those with a medium level of overall social media engagement also had a very high level of engagement.

The results obtained from data processing showed that the highest frequencies were found for average and high levels in the adsorption dimension, at 37.0% and 34.3%, respectively. Some specificities can be highlighted based on the levels of social media addiction (Table 5):

- Among students with a medium level of overall addiction to social networks, those with average (35.2%) and high (33.2%) levels of adsorption prevailed.
- However, students with an average level of adsorption and a high level of obsession with social networks (20.9%) and excessive use of social networks (26.5%) predominated.
- Likewise, respondents with a high level of adsorption and a medium level of lack of control over social network use predominated (21.2%).

Correlation analysis between the academic engagement and social media addiction variables, including their respective dimensions, confirmed their presence in all cases. The correlation was highly statistically significant (*p* < 0.01), negative, and of mild-to-moderate intensity, with Pearson r values ranging from −0.101 to −0.297. The lowest coefficient values were observed in the correlation between overall social media addiction and overall academic engagement, as well as its various dimensions (Pearson r values ranged from −0.101 to −0.130) (Table 6).

Regarding the primary use of social networks by respondents, the majority indicated leisure activities (47.8%), followed by socializing with friends and family (42.8%). In comparison, only 9.2% indicated that their most frequent use was for the exchange of scientific and technical information, with 33 students corresponding to the nursing degree. The statistical association between the main use of social networks and the degree course of the participants was significant, with a *p*-value of 0.003 (χ^2^ Pearson) (Table 7).

Students enrolled in the second, third, and sixth semesters were most likely to report sharing scientific and/or academic information as their primary use of social media, accounting for 2.4%, 2.0%, and 2.0% of the total, respectively. These two variables were also significantly associated, with a *p*-value of 0.020 obtained in the Pearson χ^2^ test (Table 7).

Among the study sample, the majority had active accounts on three social networks: Instagram (currently X), Facebook, and WhatsApp (62.6%). In addition, the majority regularly used these four to seven times a day (27.2%). Only 13.6% stated that they did not use them daily. The same situation was observed when characterizing the data by specific scale categories through the crossing of variables, establishing a statistically significant association between the two variables, with a *p*-value < 0.01 for the Pearson χ^2^ (Table 7).

When identifying the main reason for controlling their use of social media, students overwhelmingly selected the possibility of being victims of crime and bullying (41.8%); among these, those who use social media primarily to socialize with friends and family predominated (236 participants). It is essential to highlight that prioritizing academic activities and avoiding procrastination in this area was mentioned by only 17.9%. The results of the Pearson χ^2^ test (*p* = 0.033) indicated a statistically significant association between these two variables (Table 8).

When analyzing the association between degree program and the perceived main reason for controlling the use of social networks, atypical behavior was only detected in those enrolled in clinical psychology, with greater concern among students about avoiding an addiction (48 respondents), being affected by academic procrastination (41 students), and seeking more time to spend with family and friends (25 participants). Both variables were significantly associated in a statistically significant manner, with a *p*-value < 0.01 in the Pearson χ^2^ test (Table 8).

## 4. Discussion

In the context investigated in this study, in which social media addiction was found to be correlated with academic engagement, Bittencourt Spricigo et al. (2023) established that elements related to the inappropriate use of technologies constitute a barrier to the academic engagement of university students. Likewise, Shomotova and Ibrahim (2025) mention that the desired state of this variable favors the perception of educational and professional success. In another context, Gaxiola Romero et al. (2023) found a significant correlation (*p* < 0.01) between academic engagement and the subjective well-being of the students participating in their research (r = 0.32). In line with the above, Sanchez Ruiz et al. (2024) conducted a study on 717 university students from a Lebanese university, finding a significant relationship (*p* < 0.01) between academic engagement with self-care (r = 0.42), positive affects (r = 0.51), and self-perception of performance (r = 0.60). According to the results obtained by the authors of this research, this would imply that a decrease in levels of addiction to social networks could improve the variables positively associated with academic engagement.

Consistent with the results obtained in this research, with a predominance of average and high levels of academic engagement, Zamudio Elizalde (2021) found a predominance of good and excellent levels (exceeding 95% when combined) of school engagement in both its cognitive and behavioral dimensions. Likewise, García Rodríguez et al. (2022) observed very similar frequencies of medium and low levels (approximately 37% and 13%, respectively) of academic engagement, as well as its dimensions of dedication, vigor, and absorption. Likewise, Miñan Olivos et al. (2023) also reported a predominance of medium-level social media addiction in their sample of university students, with a mean score of 50.74 ± 12.7.

In the context investigated, the results showed a predominance of the average level of academic engagement, which did not coincide with what was observed by Estrada Araoz and Paricahua Peralta (2023), who also applied the UWES-S instrument, showing a higher frequency of low levels of academic engagement and its dimensions, with a rate of 23.2% and 26.8% among the participants. Thus, this suggests that the frequency values of these variables depend on the academic environment.

Garriott et al. (2023) conducted a qualitative study of 32 Latino engineering students at 11 higher education institutions in the United States. They found that a high level of academic engagement fosters a strong sense of community based on respect and the pursuit of a common goal, which can be facilitated through effective use of social media.

Another similar result was published by Arteaga Araujo et al. (2022), who also found a predominance of a medium level of social media addiction (38.8%) and the dimensions studied: obsession (38.8%), lack of control of use (40.8%), and excessive use (39.5%). In addition, they also found a significant correlation (*p* < 0.05) between this variable and academic self-regulation (r = −0.188; *p* = 0.001) and procrastination (r = 0.501; p = 0.000). In the same way, in a study involving 4852 adolescents from various regions of mainland China, Liu et al. (2022) found that problematic Internet use was negatively and significantly associated with academic engagement (β = −0.26; *p* < 0.001). Yang et al. (2022) observed a significant correlation between smartphone addiction and the level of online interpersonal interaction, as well as with the use of the Internet to share information with peers.

According to Suárez Perdomo et al. (2022), in their sample of university students, social media addiction was significantly related to academic procrastination (*p* = 0.000); 39% had a low level of addiction, and 11% had a high level. Likewise, Ramírez Gil et al. (2021) found a significant association between self-regulation and procrastination (*p* < 0.01), as well as between procrastination and solving problems using social media (*p* < 0.01). Similarly, López Angulo et al. (2021) investigated the intention to drop out of school and found that it was negatively related to academic engagement (r = −0.46; *p* < 0.001), which, in turn, was positively associated with the perception of support for academic autonomy (r = 0.43, *p* < 0.001); therefore, reducing the excessive use of social media could contribute to reducing school dropout rates, according to the results of this research.

Based on the results of this study combined with those observed in other reports published by other authors, actions aimed at preventing social media addiction can improve the status of other variables positively related to academic engagement, such as the following: Navarro Huaringa et al. (2022) determined the presence of a statistically significant (*p* = 0.000) and moderate positive relationship between the learning climate and academic engagement (r = 0.403), as well as its dimensions, namely, willingness to study (r = 0.444) and satisfaction with studying (r = 0.267). In a research study conducted in a Peruvian university context, Arredondo Salas et al. (2022) found a statistically significant positive correlation of academic engagement with academic satisfaction (r = 0.686; *p* < 0.001) and with affective engagement (r = 0.689; *p* < 0.001). Serrano et al. (2022) found a statistically significant positive correlation (*p* < 0.01) between academic engagement and basic personality traits, as well as academic achievement, with moderate intensity (r values ranging from 0.19 to 0.44). In another study by Bautista Quispe et al. (2023), the results showed a statistically significant (*p* < 0.05) and negative correlation between social media addiction and its dimensions, specifically with respect to academic self-regulation (r = −0.410).

Results consistent with those observed in the present investigation were observed by Manzur et al. (2024), who conducted a study among nursing students at an Argentine university, finding a positive, significant (*p* < 0.01), and moderate-intensity correlation between the dimensions of academic engagement, namely vigor (rho = 0.465), dedication (rho = 0.526), and absorption (rho = 0.418), and academic satisfaction. In another investigation, when analyzing the relationship between academic engagement and its dimensions (vigor, dedication, and absorption) with respect to academic procrastination and its dimensions (postponement and self-regulation), Córdova Gonzales et al. (2024) established negative correlations that were statistically significant (*p* = 0.00) and with low and moderate magnitudes (r values between −0.17 and −0.63). Likewise, Peker (2024) observed that academic engagement was statistically correlated with time management in 42 university students studying psychology and sociology (r = 0.55; *p* < 0.001).

In a sample of 194 students from Al-Diwaniyah schools, Almurumudhe et al. (2022) found a significant positive relationship between psychological capital and academic engagement, as well as between self-esteem and academic performance (*p* < 0.01). Furthermore, academic procrastination was found to have a significant negative relationship with both self-esteem and academic performance (*p* < 0.01). Likewise, self-esteem partially mediates the relationship between psychological capital, academic engagement, and academic procrastination with respect to academic performance.

Some variables affected by social media addiction benefit from adequate levels of academic engagement. In this regard, the results of a study involving 751 students from the Faculty of Education Sciences at the University of Granada, Spain, Lizarte Simón et al. (2024) found a significant, moderate, and negative correlation between homework anxiety and academic engagement (r = −0.296; *p* < 0.001). Widowati et al. (2023) established a statistically significant (*p* < 0.01) positive and moderate correlation between academic engagement and digital literacy (r = 0.690), self-efficacy (r = 0.584), and academic performance (r = 0.562).

Similar to the findings of the present research regarding the main uses of social networks by students, a study conducted in Guangdong, China, involving 591 English-language learners by Yin et al. (2023) found a significant association between social media use for entertainment and socializing and the sociobehavioral aspect of academic engagement (*p* < 0.001). The authors also observed a significant association between social media use and the cognitive and emotional aspects of academic engagement (*p* < 0.001), which corresponds to the association established in this study.

The main limitation of this study is the potential for extrapolating the results to other contexts, given the multicausal nature of the phenomena studied and the fact that the population consisted of students in health sciences programs. Therefore, it would be advisable to diversify participant composition in future research. Likewise, instead of a cross-sectional study, a longitudinal one would provide an evolutionary view of the phenomenon under investigation. The study investigates the problem through an observational design, but it would be advisable to design new research that delves into socio-educational programs, plans or strategies using an experimental design.

The results obtained have the potential to inform a situational assessment that could be used by the authorities of the National University of Chimborazo to implement effective educational strategies. Furthermore, these results will serve as a basis for future research on the main study variables. The discovery of the relationship between social media addiction and academic engagement provides further empirical evidence for the relevant entities to assess its inclusion among behavioral addictions. Furthermore, the results provide a basis for studying possible strategies to reduce school dropout rates by mitigating the excessive use of social media to strengthen academic commitment.

## 5. Conclusions

The population was predominantly composed of students with sufficient academic engagement and a moderate level of social media addiction, with both variables being significantly associated. Most participants reported an average level of academic engagement.

The levels of social media addiction and academic engagement, in addition to the corresponding dimensions, were statistically significant and negatively correlated with moderate intensity.

The results showed a significant statistical association between the social media used and the frequency of connection, as well as between the primary use of social media and the primary reason for controlling their use.

The main theoretical contribution of this study consists of identifying social media addiction as a variable capable of affecting academic engagement in university students. Thus, the results indicate that higher education institutions should implement measures to promote the appropriate use of social networks among students as part of pedagogical strategies aimed at increasing their academic engagement. The results serve as a basis for future studies and as a diagnostic basis for establishing policies and strategies in the institution where the research was conducted to increase academic engagement and reduce social media addiction.

## Figures and Tables

**Table 1:** Sufficiency of academic engagement according to the level of addiction to social networks.

Academic Engagement	Level Dimensions of Social Media Addiction						Total	
	Low		Medium		High			
	n	%	n	%	n	%	n	%
	Obsession with Social Networks [*p* = 0.000 ** (χ^2^ Pearson)]							
Insufficient	--	--	11	0.9	40	3.3	51	4.2
Enough	--	--	618	51.5	531	44.3	1149	95.8
Total	--	--	629	52.4	571	47.6	1200	100.0
	**Lack of Control Over the Use of Social Networks** [*p* = 0.000 ** (χ^2^ Pearson)]							
Insufficient	1	0.1	13	1.1	37	3.1	51	4.2
Enough	25	2.1	655	54.6	469	39.1	1149	95.8
Total	26	2.2	668	55.7	506	42.1	1200	100.0
	**Excessive Use of Social Networks** [*p* = 0.048 * (χ^2^ Pearson)]							
Insufficient	2	0.2	9	0.8	40	3.3	51	4.2
Enough	33	2.8	394	32.8	722	60.2	1149	95.8
Total	35	2.9	403	33.6	762	63.5	1200	100.0
	**Social Media Addiction (Global)** [*p* = 0.000 ** (χ^2^ Pearson)]							
Insufficient	4	0.3	32	2.7	15	1.2	51	4.2
Enough	24	2.0	1092	91.0	33	2.8	1149	95.8
Total	28	2.3	1124	93.7	48	4.0	1200	100.0

Note: ** Statistically significant at *p* < 0.01. * Statistically significant at *p* < 0.05.

**Table 2:** Level of academic engagement according to level of addiction to social networks.

Level of Academic Engagement	Level Dimensions of Social Media Addiction						Total	
	Low		Medium		High			
	n	%	n	%	n	%	n	%
	Obsession with Social Media							
Very low	--	--	3	0.2	8	0.7	11	0.9
Low	--	--	8	0.7	32	2.7	40	3.3
Average	--	--	206	17.2	280	23.3	486	40.5
High	--	--	242	20.2	169	14.1	411	34.2
Very high	--	--	170	14.2	82	6.8	252	21.0
Total	--	--	629	52.4	571	47.6	1200	100.0
	**Lack of Control Over the Use of Social Networks**							
Very low	1	0.1	2	0.2	8	0.7	11	0.9
Low	--	--	11	0.9	29	2.4	40	3.3
Average	8	0.7	222	18.5	256	21.3	486	40.5
High	6	0.5	263	21.9	142	11.8	411	34.2
Very high	11	0.9	170	14.2	71	5.9	252	21.0
Total	26	2.2	668	55.7	506	42.1	1200	100.0
	**Excessive Use of Social Networks**							
Very low	1	0.1	2	0.2	8	0.7	11	0.9
Low	1	0.1	7	0.6	32	2.7	40	3.3
Average	7	0.6	125	10.4	354	29.5	486	40.5
High	11	0.9	146	12.2	254	21.2	411	34.2
Very high	15	1.2	123	10.2	114	9.5	252	21.0
Total	35	2.9	403	33.6	762	63.5	1200	100.0
	**Social Media Addiction (Global)**							
Very low	3	0.2	1	0.1	7	0.6	11	0.9
Low	1	0.1	31	2.6	8	0.7	40	3.3
Average	6	0.5	466	38.8	14	1.2	486	40.5
High	7	0.6	397	33.1	7	0.6	411	34.2
Very high	11	0.9	229	19.1	12	1.0	252	21.0
Total	28	2.3	1124	93.7	48	4.0	1200	100.0

**Table 3:** The level of the vigor dimension, corresponding to the academic engagement variable, according to the level of addiction to social networks.

Level of Vigor	Level Dimensions of Social Media Addiction						Total	
	Low		Medium		High			
	n	%	n	%	n	%	n	%
	Obsession with Social Media							
Very low	--	--	5	0.4	20	1.7	25	2.1
Low	--	--	16	1.3	43	3.6	59	4.9
Average	--	--	288	24.0	317	26.4	605	50.4
High	--	--	193	16.1	131	10.9	324	27.0
Very high	--	--	127	10.6	60	5.0	187	15.6
Total	--	--	629	52.4	571	47.6	1200	100.0
	**Lack of Control Over the Use of Social Networks**							
Very low	1	0.1	4	0.3	20	1.7	25	2.1
Low	1	0.1	19	1.6	39	3.2	59	4.9
Average	7	0.6	303	25.2	295	24.6	605	50.4
High	6	0.5	214	17.8	104	8.7	324	27.0
Very high	11	0.9	128	10.7	48	4.0	187	15.6
Total	26	2.2	668	55.7	506	42.1	1200	100.0
	**Excessive Use of Social Networks**							
Very low	2	0.2	3	0.2	20	1.7	25	2.1
Low	--	--	10	0.8	49	4.1	59	4.9
Average	9	0.8	168	14.0	428	35.7	605	50.4
High	7	0.6	133	11.1	184	15.3	324	27.0
Very high	17	1.4	89	7.4	81	6.8	187	15.6
Total	35	2.9	403	33.6	762	63.5	1200	100.0
	**Social Media Addiction (Global)**							
Very low	4	0.3	9	0.8	12	1.0	25	2.1
Low	--	--	54	4.5	5	0.4	59	4.9
Average	7	0.6	583	48.6	15	1.2	605	50.4
High	5	0.4	312	26.0	7	0.6	324	27.0
Very high	12	1.0	166	13.8	9	0.8	187	15.6
**Total**	28	2.3	1124	93.7	48	4.0	1200	100.0

**Table 4:** The level of the dedication dimension, corresponding to the academic engagement variable, according to the level of addiction to social networks.

Level of Dedication	Level Dimensions of Social Media Addiction						Total	
	Low		Medium		High			
	n	%	n	%	n	%	n	%
	Obsession with Social Media							
Very low	--	--	1	0.1	8	0.7	9	0.8
Low	--	--	14	1.2	37	3.1	51	4.2
Average	--	--	139	11.6	257	21.4	396	33.0
High	--	--	175	14.6	124	10.3	299	24.9
Very high	--	--	300	25.0	145	12.1	445	37.1
Total	--	--	629	52.4	571	47.6	1200	100.0
	**Lack of Control Over the Use of Social Networks**							
Very low	1	0.1	--	--	8	0.7	9	0.8
Low	1	0.1	17	1.4	33	2.8	51	4.2
Average	6	0.5	159	13.2	231	19.2	396	33.0
High	4	0.3	192	16.0	103	8.6	299	24.9
Very high	14	1.2	300	25.0	131	10.1	445	37.1
Total	26	2.2	668	55.7	506	42.1	1200	100.0
	**Excessive Use of Social Networks**							
Very low	--	--	1	0.1	8	0.7	9	0.8
Low	2	0.2	13	1.1	36	3.0	51	4.2
Average	6	0.5	80	6.7	310	25.8	396	33.0
High	5	0.4	119	9.9	175	14.6	299	24.9
Very high	22	1.8	190	15.8	233	19.4	445	37.1
Total	35	2.9	403	33.6	762	63.5	1200	100.0
	**Social Media Addiction (Global)**							
Very low	1	0.1	2	0.2	6	0.5	9	0.8
Low	3	0.2	39	3.2	9	0.8	51	4.2
Average	5	0.4	378	31.5	13	1.1	396	33.0
High	5	0.4	288	24.0	6	0.5	299	24.9
Very high	14	1.2	417	34.8	14	1.2	445	37.1
Total	28	2.3	1124	93.7	48	4.0	1200	100.0

**Table 5:** The level of the adsorption dimension, corresponding to the academic engagement variable, according to the level of addiction to social networks.

Adsorption Level	Level Dimensions of Social Media Addiction						Total	
	Low		Medium		High			
	n	%	n	%	n	%	n	%
	Obsession with Social Media							
Very low	--	--	4	0.3	6	0.5	10	0.8
Low	--	--	4	0.3	21	1.8	25	2.1
Average	--	--	193	16.1	251	20.9	444	37.0
High	--	--	225	18.8	187	15.6	412	34.3
Very high	--	--	203	16.9	106	8.8	309	25.8
Total	--	--	629	52.4	571	47.6	1200	100.0
	**Lack of Control Over the Use of Social Networks**							
Very low	1	0.1	2	0.2	7	0.6	10	0.8
Low	--	--	4	0.3	21	1.8	25	2.1
Average	8	0.7	201	16.8	235	19.6	444	37.0
High	6	0.5	254	21.2	152	12.7	412	34.3
Very high	11	0.9	207	17.2	91	7.6	309	25.8
Total	26	2.2	668	55.7	506	42.1	1200	100.0
	**Excessive Use of Social Networks**							
Very low	1	0.1	2	0.2	7	0.6	10	0.8
Low	--	--	2	0.2	23	1.9	25	2.1
Average	8	0.7	118	9.8	318	26.5	444	37.0
High	9	0.8	137	11.4	266	22.2	412	34.3
Very high	17	1.4	144	12.0	148	12.3	309	25.8
Total	35	2.9	403	33.6	762	63.5	1200	100.0
	**Social Media Addiction (Global)**							
Very low	3	0.2	1	0.1	6	0.5	10	0.8
Low	--	--	18	1.5	7	0.6	25	2.1
Average	6	0.5	423	35.2	15	1.2	444	37.0
High	7	0.6	399	33.2	6	0.5	412	34.3
Very high	12	1.0	283	23.6	14	1.2	309	25.8
Total	28	2.3	1124	93.7	48	4.0	1200	100.0

**Table 6:** Correlation between academic engagement and social media addiction.

Level Dimensions of Academic Engagement	Level Dimensions of Addiction to the Use of Social Networks			
	Obsession	Lack of Control	Excessive Use	Global
Global	−0.233 **	−0.232 **	−0.214 **	−0.104 **
Vigor	−0.211 **	−0.247 **	−0.236 **	−0.130 **
Dedication	−0.297 **	−0.261 **	−0.216 **	−0.113 **
Absorption	−0.197 **	−0.218 **	−0.199 **	−0.101 **

Note: ** Statistically significant at the *p* < 0.01 level.

**Table 7:** Association of social networks used by participants with connection frequency.

Social Networks Used		Frequency of Connection to Social Networks						Total
		A Few Times a Month	A Few Times a Week	1 to 3 Times a Day	4 to 7 Times a Day	8 to 10 Times a Day or More	All the Time or Almost All the Time	
Instagram, Facebook, and/or WhatsApp	n	27	56	157	218	153	140	751
	%	2.2	4.7	13.1	18.2	12.8	11.7	62.6
Twitter (X), Facebook, and/or WhatsApp	n	1	3	5	5	5	3	22
	%	0.1	0.2	0.4	0.4	0.4	0.2	1.8
Only Instagram and Twitter	n	4	10	30	49	27	39	159
	%	0.3	0.8	2.5	4.1	2.2	3.2	13.2
Only Facebook and/or WhatsApp	n	36	26	81	54	30	41	268
	%	3.0	2.2	6.8	4.5	2.5	3.4	22.3
Total	n	68	95	273	326	215	223	1200
	%	5.7	7.9	22.8	27.2	17.9	18.6	100.0

Note: *p* = 0.003 (χ^2^ Pearson).

**Table 8:** The association between the main use of social networks and the main reason for controlling social media use.

The Main Use of Social Networks		The Main Reason for Controlling the Use of Social Networks					Total
		Socialize with Friends and Family	Leisure	Scientific–Academic Exchange	Uses of Various Kinds	Avoid Procrastinating Tasks	
Activities with friends and family	N	29	28	4	--	--	61
	%	2.4	23	0.3	--	--	5.1
I do not see any reason	N	34	52	6	--	--	92
	%	2.8	4.3	0.5	--	--	7.7
Financial possibility	N	1	2	2	--	--	5
	%	0.1	0.2	0.2	--	--	0.4
Avoiding an addiction	n	106	139	25	--	1	271
	%	8.8	11.6	2.1	--	0.1	22.6
Avoid procrastinating tasks	n	80	106	24	1	4	215
	%	6.7	8.8	2.0	0.1	0.3	17.9
Being a victim of crime	n	236	221	43	--	2	502
	%	19.7	18.4	3.6	--	0.2	41.8
Health impact	n	21	25	7	1	--	54
	%	1.8	2.1	0.6	0.1	--	4.5
Total	n	507	573	111	2	7	1200
	%	42.2	47.8	9.2	0.2	0.6	100.0

Note: *p* = 0.033 (χ^2^ Pearson).

## Data Availability

The data supporting the results are available upon request from the corresponding author. An anonymized dataset will be provided to anyone who requests it for academic purposes and agrees to a confidentiality agreement, subject to applicable ethical requirements.
